# Transactions of the Pathological Society of Philadelphia

**Published:** 1839-11-30

**Authors:** 


### MEDICAL EXAMINER.

DEVOTED TO MEDICINE, SURGERY, AND THE COLLATERAL SCIENCES.

No. 48.] PHILADELPHIA, SATURDAY, NOVEMBER 30, 1839. [Vol. II.

### TRANSACTIONS OF THE PATHOLOGICAL SOCIETY OF PHILADELPHIA.

*November 25th*, 1839.

The President, Dr. Gerhard, in the Chair.

*Case of Fibrous Tumour of the Uterus, and Dilatation of the Heart.* By Dr. Stewardson.

Ann Blake, a coloured woman, aged forty-five, was admitted into the Pennsylvania Hospital on the 2d of September, 1839. She was born at Albany, in the state of New York; and, when about nineteen years of age, came to Philadelphia, where she has remained ever since, engaged for the most part as cook in private families. She was married at about the age of twenty-five, and lost her husband about eleven years afterwards, having had but one child. According to the account of her mother, who is still living, and apparently in the enjoyment of good health, she was subject from infancy to shortness of breath, and cough, which were aggravated as she grew older. In early life, too, she was thin and delicate, so that her friends were fearful of consumption; but as she advanced in years, she became stouter and more fleshy, notwithstanding the steady increase of the cough and oppression; though constant, the difficulty of breathing was subject to marked exacerbations, coinciding with an increase of cough. Expectoration of whitish or yellowish sputa. Except the measles, smallpox, and whooping cough, she never suffered under more than one attack of severe acute disease, and that occurred about fourteen years before her death. It confined her to her bed about four weeks,—came on without evident cause,—was accompanied by severe pain in right side, and high fever, without any increase of her habitual cough. She was bled, and otherwise actively treated by Dr. Cox, who regarded the attack as a liver complaint, from which she apparently convalesced perfectly, without any marked aggravation of her asthmatic symptoms. These symptoms, however, continued as before steadily to increase, so that for many years before her death, certainly as long as eight or nine years, she was obliged to lie with her head very much elevated, and frequently to get up and open the window for breath. Oppression increased by exertion, especially the ascent of a flight of stairs, which was at times exceedingly difficult; sleep disturbed; starting at the slightest noise; flushes of heat, followed by cold sweats; palpitation of heart on exertion. Her appetite was strong; and except a feeling of weight at the epigastrium after eating certain articles, such as pastry, &c., her digestion was good. Her general health did not suffer; and she was able to continue in her employment as cook until January last, when her legs began to swell for the first time. Previous to this, she complained of swelling of the feet occasionally. In January, however, the œdema rapidly increased, extending from the feet upward; the skin of legs very tense. About this time she was seen by Dr. Meigs, sen., who found her labouring under the symptoms mentioned,—violent palpitation of the heart, strong pulse, &c. Under his care she was much relieved; but ultimately the dyspnœa became so great that she was obliged to quit work, and on the 2d of September was admitted into the Pennsylvania Hospital. At that time the dyspnœa was excessive; the anasarca universal; considerable effusion into abdomen; skin of both legs and belly remarkably tense; could not lie down to sleep, and at times was obliged to lean forward to relieve breathing; urine scanty, not more than half a pint daily, pale straw coloured, and not turbid; appetite poor; bowels costive; dizziness when she attempted to move, but no headach; countenance anxious. At first she took a mixture principally composed of squill and digitalis, which was followed by a freer secretion of urine, diminution of dyspnœa, &c. This amelioration was not permanent; the oppression returned, and the urine became scanty. A tea, composed of

Cream of Tartar ℥ss.
Bac. Juniperi ℥ss.

in a pint of water, daily, was then ordered. This increased the urine, and operated very gently on the bowels. In a few days the cream of tartar was increased to an ounce, and the following powder directed, viz.:

℞. Cream of Tartar ʒij.

Pulv. Jalapi gr. x.

to be repeated daily. By this means, five or six free, watery stools, and between two and three quarts of urine, were passed daily; the dyspnœa was much relieved, the appetite improved, œdema somewhat diminished, and strength increased, whilst at the same time she slept much better at night.

During her residence in the hospital, she was free from fever; pulse, of natural frequency; tongue whitish, moist, and chapped; complained of no pain any where, except, perhaps, across the small of the back, accompanied with a sensation of great weakness in same part, as well as in lower limbs; auscultation difficult, on account of great enlargement of mamma, &c., and was not performed with any care; a bruit de soufflet was heard,—but whether in the first or second sound, is not recollected.

No material change took place until within two or three days of her death, when the weather became cool and damp. Her oppression then increased so much that she was obliged almost constantly to lean forwards, resting her breast and head on a pillow; stools less frequent; anasarca increased so much that the skin under clavicles was puffed out for an inch; face œdematous; cellular membrane under the conjunctiva of the ball of the eye distended, of a dull white, giving to the cornea a sunken appearance, as in chymosis; some purulent discharge from right eye. She died suddenly, without stupor, on the morning of September 30th.

It should also be observed that during her residence in the hospital, there was neither leucorrhœal nor other discharge from the vagina, and that it is probable her menstrual discharge had been arrested for a year or more, as she attributed her attack in January to change of life.

*Autopsy*, *Oct.* 1*st.*—Tissue of skin over abdomen, where alone it was examined, very dense and firm; cellular tissue beneath, and also that covering the walls of the chest anteriorly, dense, thickened, and of an opaque white; meshes distended with fluid, so that the whole thickness of the cellular layer is from one to two inches; abdominal muscles thinned.

*Abdomen.*—Several quarts of clear yellowish fluid in its cavities.

*Liver* extending several inches below margin of false ribs, on right side, and in front; not much enlarged, however; tissue very firm, less easily penetrated than natural; the cut surface is rough and granulated, and so firm that it produces a grating sound when the handle of the knife is drawn across it; two substances very distinct; lobules more defined than common; central point reddish, surrounded by a yellow fawn-colour; the interlobular structure exceedingly dark, almost venous black.

*Kidneys* normal.

*Spleen* very small, firm.

*Bladder* very much contracted, containing no urine.

Portions of the *intestine*, especially the rectum, and part of colon, very much contracted in their circumference,—the latter being about the size of a healthy aorta, or somewhat less. The mucous membrane of the small intestine presents nothing remarkable; peritoneal covering thickened, and more opaque than natural.

*Stomach* of moderate size; its walls thick, owing to hypertrophy of muscular coat; mucous membrane of a pale, delicate rose tint, of natural consistence and thickness.

*Chest.*—Large effusion of serum into cavity of both pleuræ; strong adhesion between base of right lung and diaphragm, but few elsewhere on same side; none at left.

*Right lung* rather smaller than natural, supple, crepitating, most firm in inferior lobe, where it is of a deep brown colour when cut into and congested; no tubercles.

*Left lung* not more than half its natural size, supple, and crepitating throughout; vesicles dilated anteriorly, and along from border; tissue otherwise healthy; no tubercles.

*Pericardium* contains a few ounces of fluid; no adhesions. Heart about twice its natural size; walls firm; right ventricle much enlarged,—would contain a goose's egg,—measures four and a half inches from point to origin of pulmonary artery; breadth across base two and a quarter inches from septum; columnæ corneæ very firm and thickened. Independent of these, the walls measure five lines in thickness, except at the point, where they are from two to three lines. Lining membrane smooth and natural; circumference of auriculo-ventricular orifice near five inches. Tricuspid valve somewhat thickened, more or less opaque; right auricle considerably dilated; columnæ corneæ rather thickened; internal membrane smooth, somewhat opaque, and thickened; left ventricle but slightly, if at all, enlarged; lining membrane smooth and natural,—length three and a half inches,—breadth from septum one and three-quarter inches at base; thickness of walls six lines; mitral valve considerably thickened, especially along margin, where it is from one to two lines, one of its lolds very much contracted.

*Left auricle* about natural size; lining membrane smooth, more or less opaque, thickened, divisible into layers.

Circumference of auriculo-ventricular orifice three and three-quarter inches.

*Aortic* and *pulmonary valves* natural, except that the former are a little thickened.

The length of the heart along the septum is four and a quarter inches, and its circumference at base ten inches, after the heart had been opened, and had remained macerating for a day or two.

*Aorta* presents nothing remarkable, except numerous cartilaginous deposits under its lining membrane, and one which is ossified.

*Generative organs.*—The body of the uterus is very much enlarged. The whole length of the organ from fundus to os tincæ is four and a half inches; near the fundus, its breadth and thickness are each three and a half inches. Its form is exceedingly irregular, its external surface being studded with a number of protuberances, varying in size from that of a small marble to an English walnut. Their bases are generally broad, but in some few they are very contracted, thus having the appearance of distinct rounded masses attached to the womb by a narrow peduncle. The colon is uniformly covered by the peritoneal membrane, smooth polished, but thickened. When cut into, these protuberances are found to consist principally of a rounded mass, very dense and firm, creaking somewhat under the scalpel, of a striated and areolar structure, of a dead white colour, perfectly distinct from the surrounding parts, from which it is separated by a cellular cyst. Surrounding this, even in those which are pedunculated, is a thin layer of the proper substance of the womb, not materially altered in character. Carrying the incision completely through the walls into the cavity of the uterus, a number of bodies similar to those above described, but mostly smaller, are found imbedded in its substance, which is of a bright red colour, distinctly striated, of about natural consistence, being merely hypertrophied.

Thickness of walls near fundus one and a half inches, tapering to an inch near neck.

Cavity of uterus about two inches in length; its internal surface irregular, owing to the protuberance of the masses above described. It contains a coagulum one and a half inches long, by six lines broad at its base, tapering to a point, and apparently composed of lymph, mixed with a few red globules, and firmly adhering to internal surface of uterus at its upper part. Neck of uterus rounded, projects four lines into the vagina, and is ten lines broad, smooth, and without irregularity of surface; os tincæ circular, and will scarcely more than admit the introduction of a large probe.

The *vagina* presents nothing remarkable.

*Recapitulation.*—A female, of rather thin and delicate make, is subject from her earliest infancy to cough and oppression, which become steadily worse as she advances in life, and yet, after arriving at the age of womanhood, she becomes more stout and fleshy,—and, notwithstanding the continually increasing severity of her habitual symptoms, she enjoys in other respects good health until near the close of her life. Except the usual infantile diseases, she suffers under but one attack of acute disease, supposed to be liver complaint, with which she is affected about fourteen years before her death, and which is apparently perfectly cured at the expiration of a few weeks, and occasions no subsequent inconvenience. For some years before her death, the dyspnœa, always subject to exacerbation coinciding with an increase of cough, becomes excessive, and is accompanied with palpitations. Finally, about nine months before her death, œdema of the low extremities, preceded for some time by slight œdema of ankles, comes on, and rapidly extends itself to the abdomen, and finally becomes general. The effusion is much lessened for a time by the use of purgatives and diuretics,—but, at last, under the influence of cold, becomes suddenly very great, and the patient dies in a state of great oppression, without stupor.

On examining the body after death, one lung is found partly emphysematous; considerable serous effusion exists in the cavities of the pleuræ and peritoneum, as well as the cellular membrane generally; the right ventricle of the heart very much hypertrophied and enlarged, and the auriculo-ventricular orifice of the same side dilated; the liver indurated, owing to the altered character of the acini, which are not only firmer, but more prominent, and paler than natural, with a yellow tinge; the womb much enlarged, containing a number of bodies presenting more or less the characters of fibrous tumours.

*Remarks.*—That the early symptoms in this case depended upon emphysema of the lungs, can scarcely admit of doubt; for the paroxysmal dyspnœa existing from infancy, accompanied merely by cough, and without disturbance of any of the other functions, is quite characteristic. It is perfectly evident, moreover, that the effusion into the chest and the dilatation of the heart, the only other conditions which could account for the oppression, came on at a much later period, and, consequently, however much they might have aggravated it towards the close of the patient's life, could have had nothing to do with causing it at an earlier period. But it may be asked, if we attribute the cough and dyspnœa to the dilatation of the pulmonary vesicles, why was the latter lesion not found more extensively, and to a more marked degree? Had the lungs been more minutely examined with reference to this point, I think it probable that evidence of greater dilatation would have been found. But the most conclusive answer to the objection will probably be found in the fact that the large effusion of serum, by which the right lung was reduced to about half its normal size, and which had existed perhaps for six or nine months previous to death, had, by compression, restored the cells, in part, to their natural dimensions. I am not aware that the effect of this kind of compression upon an emphysematous lung has any where been noted; and, indeed, the existence of hydrothorax is not mentioned by Dr. Louis as having existed in any of the numerous cases lately analyzed by him in his treatise on emphysema. Nevertheless, although we cannot appeal to experience to settle the question, the supposed effect of the compression is in itself so reasonable, and accounts so perfectly for the appearances observed, as to render it highly probable.

In accordance with what is so commonly observed in cases of emphysema of long continuance, the heart was found enlarged. If we suppose this enlargement to be dependent upon the dyspnœa, and consequent obstruction to the pulmonary circulation, we should expect to find it principally upon the right side, and such was the fact in the present instance, the right cavities, and especially the right ventricle, being almost exclusively the seat of dilatation, which was particularly remarkable towards the origin of the pulmonary artery.

It can hardly be a question whether the disease of the heart, or that of the liver, was the cause of the dropsical effusion,—which commenced in the lower extremities, and extended upwards, the abdominal cavity being implicated secondarily, and that of the pleura being also involved. These circumstances are exactly in accordance with what we observe in dropsical effusion arising from disease of the heart. Those, on the contrary, which depend upon disease of the liver, almost always commence in the cavity of the peritoneum, and are not unfrequently, if we except a slight œdema of the extremities, confined to that part. It is easy to conceive of the manner in which the serous effusion in the present instance was occasioned by the disease of the heart, if we recollect that the right auriculo-ventricular orifice was dilated, and must consequently have admitted of regurgitation of blood into the auricle and vena cava, thus causing a direct obstruction to the venous circulation.

The alteration of the liver is particularly interesting, when considered in connection with the previous attack of inflammation of its substance, or in its immediate neighbourhood, under which the patient had laboured about fourteen years before her death. That the pleura was involved in this inflammation, is probable from the old adhesion which existed between the base of the right lung and diaphragm. I am disposed to attribute the alteration of the liver to the previous inflammation, for two reasons. 1st. Because it is the only known cause to which it could be attributed, if we except the disease of the heart, which probably came on too near the fatal termination to be itself the cause of a change of structure, requiring, no doubt, a considerable length of time for its production. Besides, I am not aware that disease of the heart is at all productive of the particular change in question.

2d. Because I believe that in all cases where I have observed this particular condition, I have been able to trace it to inflammation, either of the liver or the parts in its immediate neighbourhood. As I shall soon have an opportunity of again alluding to the character and nature of this alteration, as well as its influence in the production of abdominal dropsy, I will not detain the Society longer at present.

The cause of death in the patient before us must clearly be looked for in the great and sudden increase of effusion into the different cavities and cellular membrane, by which the action of the corresponding organs, and especially the lungs, was much impeded, thus occasioning the most inexpressible anxiety. Of the disease of the womb I will merely observe, that it gave rise to no symptoms, except pain in the small of the back and loins, which could not have been very severe, as the patient never called my attention to them.

